# A pilot study of a longitudinal mindfulness curriculum in undergraduate medical education

**DOI:** 10.36834/cmej.56726

**Published:** 2020-08-06

**Authors:** Heather MacLean, Emelie Braschi, Douglas Archibald, Millaray Sanchez-Campos, Danusha Jebanesan, Diana Koszycki, Carol Gonsalves

**Affiliations:** 1 Division of Neurology, Department of Medicine, University of Ottawa, Ontario, Canada; 2 Department of Family Medicine, University of Ottawa, Ontario, Canada; 3 Faculty Medicine, University of Ottawa, Ontario, Canada; 4 Faculty of Education, University of Ottawa, Ontario, Canada; 5 Division of Hematology, Department of Medicine, University of Ottawa, Ontario, Canada

## Abstract

**Background:**

To support student well-being, a mindfulness curriculum in undergraduate medical education was launched at our university in 2014. We describe the program and report 3-year results.

**Methods:**

Medical students responded to online questionnaires on mindfulness (Freiburg Mindfulness Inventory), empathy (Jefferson Scale of Physician Empathy), resilience (Connor-Davidson Resilience Scale) and perceived stress (Perceived Stress Scale) and were surveyed for demographics, home practice, and subjective experience at curriculum launch and yearly for 3 years.

**Results:**

In respondents, high stress (19.2 (SD=6)) and low resilience (71.2 (SD=12.5)) scores were seen throughout training. Scores for mindfulness correlated positively with those for empathy (r=.217 *p* < 0.01) and resilience (*r* = .539, *p* < 0.01), and negatively with stress scores (*r* = -.380, *p* < 0.01). While overall scale scores did not statistically change after curriculum implementation, statistically significant increases were seen in mindfulness (12%, *p* = 0.008), empathy (5%, *p* = 0.045), and resilience scores (12%, *p* = 0.002) with a trend toward lower stress scores (8%, *p* =0.080) in respondents who felt they applied the curriculum principles. Two hours of reported home practice per week was associated with statistically significant changes (14% increased mindfulness scores *p* < 0.001; 6% increased empathy scores *p* < 0.001, 10% increased resilience scores *p* = 0.003; 11% decreased stress scores *p* = 0.008). Despite positive program evaluations for both mandatory and elective sessions, student attendance at elective sessions was low.

**Conclusion:**

A mindfulness curriculum integrated into formal undergraduate medical education is feasible. Benefits may be confined to those students who apply curriculum principles and practice regularly. Further study is needed.

Résumé

**Contexte:**

Pour soutenir le bien-être des étudiants, un cursus de méditation pleine conscience dans le cadre du programme de doctorat en médecine a été lancé à notre université en 2014. Nous décrivons le programme et communiquons les résultats après trois ans.

**Méthodes:**

Des étudiants en médecine ont répondu à des questionnaires en ligne sur la pleine conscience (inventaire de pleine conscience de Freiburg), l’empathie (Échelle de Jefferson sur l’empathie du médecin), la résilience (échelle de résilience de Connor-Davidson) et le stress perçu (échelle du stress perçu) et ont été interrogés pour les caractéristiques démographiques, la pratique de la méditation pleine conscience à domicile et l’expérience subjective au moment du lancement du cursus et, ensuite, annuellement pendant trois ans.

**Résultats:**

Chez les répondants, un degré de stress élevé (19,2 (ET=6)) et une faible résilience (71,2 (ET=12,5)) ont été observés tout au long de la formation. Il y avait une corrélation positive entre les la pleine conscience et l’empathie (r=0,17, p<0,01) et de la résilience (r=0,539, p<0,01), et négative avec le stress (r=-0,380, p<0,01). Alors que les résultats globaux ne changent pas statistiquement après la mise en œuvre du cursus, des augmentations statistiquement significatives ont été observées pour la pleine conscience (12%, p=0,008), l’empathie (5%, p=0,045) et la résilience (12%, p=0,002) avec une tendance vers des résultats inférieurs pour le stress (8%, p=0,080) chez les répondants qui estimaient avoir appliqué les principes du programme. On retrouvait des changements significatifs chez les répondants qui rapportaient avoir pratiqué deux heures par semaine à domicile (résultats accrus de 14% pour la pleine conscience (p<0,001), de 6% pour l’empathie (p<0,001) et de 10% pour la résilience (p= 0,003), et réduits de 11% pour le stress (p=0,008)). Malgré des évaluations de programme positives pour les sessions obligatoires et optionnelles, l’assiduité des étudiants aux sessions optionnelles était faible.

**Conclusion:**

Un cursus de méditation pleine conscience intégré dans des études formelles de doctorat en médecine est faisable. Les avantages peuvent être confinés aux étudiants qui appliquent les principes du programme et qui le pratiquent sur une base régulière. D’autres études sont requises.

## Introduction

Medical students are known to suffer from degrees of stress, burnout, depression, and anxiety far beyond the levels of students in other professions.^1,2^ The decline in mental health appears within the first year of medical school training and has been shown to persist through to training completion.^3,4^ Unfortunately, interventions implemented to date, including the reorganization of medical school and residency training to reflect a learner and patient-centered approach, as well as duty hour restrictions, have not produced the anticipated and needed improvements in physician and trainee stress and burnout.^2^ The cumulative effects of increased financial burden of medical training, rapid changes in the management of acute and chronic illness, and an aging patient demographic have produced a system where physicians and trainees deal with increasing demands not only on their mental and physical well-being, but on their emotional health. It is unlikely that these demands will lessen over time.^5^ While awaiting system-level changes addressing these issues, it is desirable that medical students be explicitly taught a foundation of skills for stress management, resilience, and self-awareness in order to prevent, identify, and reverse negative stress-related outcomes that may arise during medical school, residency, and beyond.^6^ Despite this fundamental need, very little core curriculum in medical education at most institutions is dedicated to fostering these self-care skills.^7^

Additionally, empathy is a quality widely studied in the medical student population. Not without debate, empathy has been shown to decrease in some medical student populations from their first year of study through to their last year despite the increased interaction with and responsibility for patients as training advances.^8,9^^,10^ Empathy is a crucial component of an effective physician-patient relationship. Higher degrees of empathy have been associated with improved physician satisfaction, clinical competence and a reduction in lawsuits^11–13^ such that any substantial risk to its nurturing is notable. Skills to preserve and enhance physician empathy and wellness should be learned well before entry into independent medical practice.

While there may be many ways to support medical student wellbeing and nurture empathy, the practice of mindfulness is worthy of exploration. Mindfulness may be defined as the practice of paying purposeful attention to present moment experiences with the particular qualities of curiosity and compassion in a non-judgmental fashion.^14^ Its origins are in eastern traditions but practice has expanded globally as well as into the realm of healthcare. The underlying tenet of mindfulness suggests that by becoming aware of our own thoughts, emotions, and experiences, we can relate to them differently and cultivate more adaptive responses rather than relying on more habitual, perhaps maladaptive, reactions.

The cultivation of mindfulness involves mental training through both formal meditation and informal mindful awareness. Regular mindfulness practice has been shown to decrease rumination and distress, and foster compassion for self and empathy towards others.^15–17^ Furthermore the benefits of mindfulness practice have been seen despite variations in a practitioner’s interest or enthusiasm about the concept of mindfulness itself; that is to say, it is the practice that is important.^18^ Studies have shown mindfulness to have positive effects on stress, burnout, and resilience in the general public^19^ and in individuals with physical and psychological morbidities.^20,21^ However, results of mindfulness-based interventions in medical education have been mixed.^22^ In mindfulness practitioners and in preliminary studies of premedical and medical students, mindfulness has been associated with higher degrees of empathy, compassion, and altruism.^17,28^ A few studies involving physicians and medical students have shown mindfulness training and practice to have short-term positive effects on stress and burnout measures but studies showing long-term benefits are scarce.^16,17,23–27,55^

Given these data and a desire to foster well-being throughout medical training, the University of Ottawa undergraduate medical school program established a Mindfulness Curriculum Working Group which developed and, in 2014, implemented a longitudinal mindfulness curriculum. We designed an associated study to explore three primary questions:

1. How do measures of mindfulness correlate with measures of empathy, resilience, and perceived stress in our learners? These factors were chosen because of their potential impact on physician wellbeing and the physician-patient relationship.
2. How do measures of mindfulness, empathy, resilience, and perceived stress compare cross-sectionally across the four years of training at the University of Ottawa undergraduate medical program? Knowledge of these trends, and particularly of potentially high-risk periods as defined by low empathy and resilience, and high perceived stress, may help identify the most effective times in the curriculum for offering wellness interventions.
3. Will a mindfulness curriculum affect medical students’ measures of mindfulness, empathy, resilience, and perceived stress? If so, this may suggest a role for such a curriculum to foster student well-being.

This paper describes the developed curriculum (structure and content), the results of our three-year study, and implications for those implementing such interventions in their own contexts.

## Methods

Our research team included faculty at the Ottawa Hospital, Bruyere Hospital, and a medical student, and family medicine resident from the University of Ottawa. Research Ethics Board approval was obtained from both the Ottawa Hospital Research Ethics Board and the Bruyère Research Ethics Board. Participation was voluntary and anonymous. Participants gave consent.

### Curriculum development and program description

In the fall of 2012, one of our group (M.S.-C.) implemented a mandatory mindfulness workshop in clerkship at the University of Ottawa. It was conducted in small groups (15 students per group in the Anglophone stream and six students per group in the Francophone stream) and took place during the Family Medicine rotation. The workshop consisted of a PowerPoint presentation reviewing data that support the benefits of mindfulness in clinical practice followed by an experiential session where students were guided through sitting, walking, and eating meditations; an exercise on mindful communication (listening and speaking); and a body scan meditation.

That same fall, we created a working group comprised of individuals in the Faculty of Medicine engaged in areas of humanities, faculty wellness, and medical education along with student representatives (the Mindfulness Curriculum Working Group). The goal of this group was to create a longitudinal mindfulness curriculum that would start in the first year of medical school, run through to clerkship, and integrate with the clerkship workshop. There was a total of 17 committee members, 10 faculty and seven students. Seven members were experienced meditators with established mindfulness practices, while 10 had more recent interest and experience. The committee used three certified mindfulness instructors as resources. Our group met every month for two academic years.

We conducted a brief grey literature review of the existing mindfulness programs within medical education in North America and internationally.^29^ Decisions regarding mandatory and elective components, curriculum objectives, content, length of sessions, timing of sessions within the mainstream curriculum, methods of delivery, and student assessment were made by consensus at our monthly meetings. The lead author (H.M) created an innovative course book on mindfulness in medicine with input from the working group members,^30^ available as a free download from Apple iBooks or in PDF format (https://books.apple.com/ca/book/mindfulness-for-medical-school-residency-and-beyond/id914285826). The lead author (H.M.) also created a faculty manual to serve as a tutor guide for teachers recruited to lead the sessions. Teachers were medical doctors and allied healthcare practitioners (including psychologists, social workers, occupational therapists), along with a neuroscientist and an associate professor of information studies. All had personal mindfulness practices greater than seven years’ duration, training in MBSR, and/or other mindfulness-based interventions. Several have taught mindfulness meditation to the university faculty and to the public. An associated curriculum website was created, accessible through the student portal, containing further reference material and related links.

The longitudinal curriculum was launched in the fall of 2014 for both the Anglophone and Francophone streams at the University of Ottawa with full support from the Vice Dean, the curriculum content review committee, the undergraduate curriculum committee, and associated student committee representatives. The curriculum incorporated both mandatory and elective components. In the mandatory sessions, students were not required to participate in the mindfulness exercises, but session attendance to at least observe the practice was expected. The curriculum started with a mandatory thirty-minute introductory session (large group, whole class) that occurred in the students’ first week of study in Year 1 of medical school. Seven additional one-hour elective sessions were offered throughout Years 1 and 2 of medical school. These again were offered as large group, whole class sessions. The structure of sessions was influenced by the style used in the Mindful Practice curriculum at the University of Rochester School of Medicine and Dentistry (https://www.urmc.rochester.edu/family-medicine/mindful-practice.aspx). Sessions were given over lunchtime.

Each session began with a mindfulness meditation: a brief sitting meditation establishing a sense of presence by focusing on the breath, bodily sensations, or other present-moment experiences. This was followed by large group learning activities with interactive and experiential components based on specific chapters from the course book. Each session focused on one mindfulness concept linked with one mindfulness skill. Topics and concepts including the ego, the voice in the head, dealing with emotional pain, stress reduction, and resilience were covered. The experiential exercises included formal meditations such breath-awareness, body-awareness, awareness of thoughts, awareness of emotion, and communication exercises among others. Every session incorporated a debrief group discussion pertaining to the concepts, exercises, and student experience. Students were given experiential assignments to practice at home between sessions along with readings from the course book. Each session ended with a short poem or koan (Zen parable) relevant to the session topic. PowerPoint slide decks for each session were developed and were available as guides for the teachers for the pre-clerkship component of the curriculum.

The mandatory half-day mindfulness workshop was then delivered in Year 3 of medical school. Table 1 summarizes the curriculum sessions.

**Table 1:** Curriculum overview

Session Title	Sample objectives
Introduction to Mindfulness (1/2 hour)	Define mindfulness List several potential benefits of mindfulness during medical training, medical practice and everyday life
The Ego & Mindful Communication (1 hour)	Describe how reflexive emotional reactions can disturb communication Illustrate mindful communication
The Voice in Head & Instantaneous Mindfulness (1 hour)	Describe automatic pilot List several ways in which one might insert moments of mindfulness into daily life
Emotional Pain & Mindfulness in Minutes (1 hour)	Describe the interaction between thoughts and emotions Define meditation
Expanding your Mindfulness Practice (1 hour)	List ways in which one might expand one’s mindfulness practice in duration and scope
Reducing Stress (1 hour)	Describe the functions of the sympathetic and parasympathetic nervous system and their associated bodily sensations/emotions when activated Demonstrate exercises that can activate the parasympathetic nervous system and promote stress reduction
Training the Brain (1 hour)	List a few structural and functional brain changes that have been observed with mindfulness practice Explain the concept of the “beginner’s mind” and potential benefits to problem solving and creativity
Resilience (1 hour)	Define equanimity Define resilience and illustrate how mindfulness might enhance it
Mindfulness Workshop (3 hours)	Practice and apply self-awareness

In the fall of 2015, regular opportunities to participate in weekly elective 30-minute guided “meditation drop-in” sessions were incorporated into the pre-clerkship curriculum. Additionally, an online mindfulness meditation program, available to students from all four years, was developed that students could access at home. In the fall of 2016, after consultation with the vice dean, the faculty of medicine undergraduate curriculum committee, and student representatives, the previously elective seven pre-clerkship sessions became mandatory for incoming medical students. The drop-in sessions remained elective.

### Population

We invited all students enrolled across the four years of training to complete the online surveys in the fall of 2014 (671 students, at curriculum launch), the spring of 2015 (671 students, one academic year post curriculum implementation), the spring of 2016 (669 students, two academic years post curriculum implementation), and the spring of 2017 (665 students, three academic years post curriculum implementation). As an incentive, participants completing surveys at each time point were eligible for a gift card draw valued at $200.

### Outcome measures

For each online survey period, we asked students to complete validated scales of mindfulness, empathy, resilience, and stress, in addition to being surveyed for demographic information and subjective experience of the curriculum. Surveys took an estimated 20-25 minutes to complete.

The short version of the Freiburg Mindfulness Inventory (hereafter referred to as the “mindfulness scale”) is a 14-item test rated on a 4-point scale (1-4; maximum of 56) that is designed to measure levels of mindfulness. Higher scores reflect higher levels of mindfulness. In one study of faculty trained as facilitators for a mindfulness course, the average was 42 (SD=5).^31^ This scale exhibits strong internal consistency α = 0.86.^32^

The Jefferson Scale of Empathy (S version; hereafter referred to as the “empathy scale”) is a 20-item test rated on a 7-point scale (1-7; maximum of 140) that is designed to measure empathy in medical students. Empathy is defined as an attribute that involves “understanding (as opposed to feeling) of the patient’s experiences, concerns and perspectives, and a capability to communicate this understanding. An intention to help by preventing and alleviating pain and suffering is an additional feature of empathy in the context of patient care.”^33–35^ Higher scores reflect higher levels of empathy. Internal consistency reliability of this version was α = 0.89 for medical students and α = 0 .87 for medical residents.^36^ Mean scores of 116 (SD=10) for female and 112 (SD=11) for males have been published from the USA, with suggested lower cut-offs of 100 for females and 95 for males.^37,38^

The Connor-Davidson Resilience Scale (hereafter referred to as the “resilience scale”) is a 25-item test rated on a 5-point scale (0-4; maximum 100) with higher scores reflecting greater resilience. This scale has documented high internal consistency and test-retest reliability.^39^ Cronbach’s alpha for the full scale was 0.89 for the general population.^47^ In a study of first year medical students the mean score was 80 (SD=9)^40^ and the population norm has been reported as 80.4.^47^

The Perceived Stress Scale (hereafter referred to as the “stress scale”) is the most widely used psychological instrument for measuring the perception of stress.^41,42^ We used the reduced 10-item scale. Items are rated on a 5-point scale (0-4; maximum of 40) with higher scores reflecting greater perceived stress. A principal components analysis revealed this scale to have an internal reliability α = 0.78.^43^ A cohort of Canadian medical students was shown to have a mean stress score of 17 (SD=6) for females and 15 (SD=6) for males^46^. The population norm has been reported as 13 and 14.2 specifically for the age group 18-29.^43^

### Data analysis

We performed comparative analyses using the demographic data and analyzed the mean scores for each of the outcome measures using independent samples t-tests and bivariate correlations. Statistical analysis was facilitated using SPSS version 23.0.

## Results

On average, 10% of Year 1 and Year 2 students attended their respective elective curricular sessions.

Students submitted a total of 378 surveys. 316 surveys were included in the analysis. This number represents a 12% overall survey response rate for the entire cohort of students enrolled in our medical school over the three years of the study (almost 2700). The majority (73%) of the respondents were from the first two years of medical training, resulting in a response rate of 18% for pre-clerkship students and 6% for clerkship students.

***Excluded entries***: We considered surveys to be complete if, for all four of the validated scales, 75% of the questions had been answered. Forty-nine entries were incomplete and therefore excluded from the analysis leaving a total of 329 surveys. During data coding, prior to the analysis, it was realized that answers to some surveys lacked the expected variability, an indication of motivation bias. Thirteen surveys showing signs of motivation bias (either when no unique answer was chosen for at least two full surveys or when non-reverse answers were chosen for reverse questions) were excluded.

***Included entries****:* A total of 316 surveys were included in the analysis. If for a given scale, one answer was missing, the score for that scale was not used for the analysis but the scores from the three other complete scales were used.

Since no identifying information was kept, it is unknown whether all 316 entries represent different students or whether some students responded at more than one study timepoint. For the statistical analysis, these entries were assumed to be independent, and will be referred to as “students” thereafter. The characteristics of the students for all four surveys are highlighted in Table 2.

**Table 2:** Respondent characteristics

		Fall	Spring		
		2014	2015	2016	2017
*N*		75	79	83	79
Age: *N* (%)	<22 22-25yo 25-30yo >30yo	14 (18.7) 45 (60.0) 10 (13.3) 4 (5.3)	19 (24.1) 51 (64.6) 7 (8.9) 2 (2.5)	2 (2.4) 56 (67.5) 22 (26.5) 3 (3.6)	5 (6.3) 65 (82.3) 8 (10.1) 1 (1.3)
Gender: *N* (%)	M F	14 (18.7) 61 (81.3)	22 (27.8) 57 (72.2)	31 (37.3) 52 (62.7)	28 (35.4) 51 (64.6)
Year of study: *N* (%)	Yr1 Yr2 Yr3 Yr4	31 (41.3) 18 (24.0) 19 (25.3) 7 (9.3)	56 (70.9) 14 (17.7) 6 (7.6) 3 (3.8)	19 (22.9) 29 (34.9) 18 (21.7) 17 (20.5)	37 (46.8) 28 (35.4) 8 (10.1) 6 (7.6)
Stream	English French	52 (69.3) 23 (30.7)	77 (97.5) 2 (2.5)	79 (95.2) 4 (4.8)	70 (88.6) 9 (11.4)
Intended specialty (multiple choices allowed)	Medical Surgical Other / Don’t know	59 (78.7) 15 (20) 19 (25.3)	57 (72.2) 22 (27.8) 13 (16.5)	71 (85.5) 16 (19.3) 13 (15.7)	68 (86.1) 16 (20.3) 16 (20.3)
Previous experience of mindfulness: *N* (%)	None Very little Some A lot	7 (9.3) 36 (48.0) 27 (36.0) 5 (6.7)	8 (10.1) 34 (43.0) 35 (44.3) 2 (2.5)	7 (8.4) 29 (34.9) 43 (51.8) 4 (4.8)	4 (5.1) 35 (44.3) 36 (45.6) 4 (5.1)
Home mindfulness practice: *N* (%)	<10min 10-60min >1-2hr >2hr	42(56) 27 (36.0) 4 (5.3) 2 (2.7)	38 (48.1) 25 (31.6) 8 (10.1) 8 (10.1)	43 (51.8) 26 (31.3) 9 (10.8) 5 (6.0)	36 (45.6) 29 (10.1) 8 (10.1) 6 (7.6)

Using data from the largest cohort of students at the same level of training (i.e. Year 1 students surveyed in the spring of 2015, 2016, and 2017 respectively), the mean mindfulness score was 37.5 (SD=6.8, *N* = 108), the mean empathy score 111.4 (SD= 13.2, *N* = 110), the mean resilience score 71.2 (SD=12.5; *N* = 110), and the mean stress score 19.2 (SD=6; *N* = 111). There were statistically significant differences between males and females for empathy (5% higher in females, 113.7 (SD=11.1) vs 107.4 (SD=15.6); *p* = 0.026), and stress (6% higher in females, 20.1 (SD=6.4) vs 17.8 (SD=5.1); *p* = 0.037).

**Figure 1:**
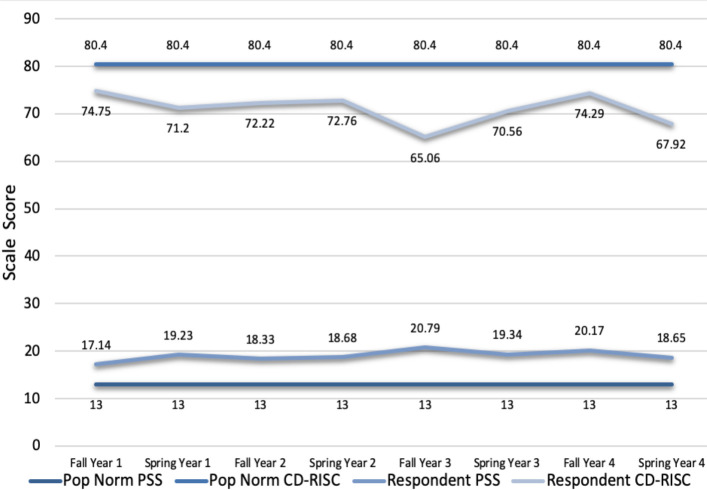
Resilience (CD-RISC) and stress (PSS) scale scores across the years of training compared to population norms^4^

Scores from the scales were compared across the years of medical training. Comparing respondents at the beginning of Year 1 of medical school to respondents at the end of Year 4 of medical school, there was no statistically significant difference in levels of mindfulness (35.2 (SD=7.6) vs 34.4 (SD 7.8), p=0.685). Empathy was 8% lower in students at the end of Year 4 (118.6 (SD 8.2) vs 107.8 (SD 16.0 p=0.004). Resilience was 7% lower (74.7 (SD 10.1) vs 67.9 (SD 14.3), p=0.050), and there was no meaningful difference in stress levels (17.1 (SD 5.9) vs 18.7 (SD 5.6), p=0.335). Stress scores remained high (range 17.1-20.8) and resilience scores relatively low (range 65.1-74.8) over the four years of medical school training. Figure 1 shows resilience scale (CD-RISC) and stress scale (PSS) scores across the years of training compared to population norms.^43, 47^

We then explored the relationship between the different scales to identify any correlations. The strongest correlations were between mindfulness and resilience scores (Pearson coefficient 0.539, *p* < 0.001) and between resilience and stress scores (Pearson coefficient -0.422, *p* < 0.001). Mindfulness scores were moderately negatively correlated with stress scores (Pearson coefficient of -0.380, *p* < 0.001). Empathy scores were weakly to moderately correlated with resilience scores (Pearson coefficient 0.286, *p* < 0.001), mindfulness scores (Pearson coefficient 0.217, *p* < 0.001), and stress scores (Pearson coefficient -0.151, *p* < 0.001).

We investigated the overall effect of the longitudinal mindfulness curriculum using surveys from medical students at the end of Year 2 of medical school. By that point in their training, the students from the MD 2017 cohort (*n* = 14) had received an introductory lecture only and were considered not to have been exposed to the curriculum. The students from the MD 2018 and 2019 cohorts (*n* = 56,) had received the same introductory lecture but also had the option of taking an additional seven elective sessions over Years 1 and 2 of medical school. Despite this opportunity for increased exposure to the curriculum in the latter two cohorts, there were no statistically significant changes in mindfulness (36.6 (SD 7) vs 36.9 (SD 6.5); *p* = 0.917), empathy (120.9 (SD 8.7) vs 115.9 (SD 9.9); *p* = 0.095), resilience (69.7 (SD 7.7) vs 73.5 (SD 11.1); *p* = 0.159), or stress (19.9 (SD 6.8) vs 18.4 (SD 5.7); *p* = 0.466) compared to the MD 2017 cohort.

Despite the inability to identify a global effect of curriculum exposure on measures of mindfulness, empathy, resilience or stress, when students who felt that they had applied the mindfulness principles taught in the curriculum were compared to students who felt that they had not applied the curriculum, there was a statistically significant increase in mindfulness scores (12% increase, 34.0 (SD 6.6) vs 40.9 (SD 9.2) *p* = 0.008), empathy scores (5% increase, 111.8 (SD9.3) vs 118.2 (SD 11.3) *p* = 0.045), resilience scores (12% increase, 69.6 (SD 9.7) vs 81.3 (SD 12.7) p=0.002) and a trend towards decreased stress scores (8% lower, 19.7 (SD 5.9) vs 16.7 (SD 5.9) *p* = 0.080). In addition, increased mindfulness practice at home was associated with proportionate increases in mindfulness, empathy and resilience scores and a proportionate reduction in stress scores which reached statistical significance for all scales for those students who reported practicing at least two hours of home mindfulness per week (14% increase in mindfulness scores 34.9 (SD 6.6) vs 42.5 (SD8.1) p<0.001; 6% increase in empathy scores 112.7 (SD 11.9) vs 121 (SD 7.8), *p* < 0.001, 10% increase in resilience scores 69.1 (SD12) vs 78.7 (SD12), *p* = 0.003; 11% decrease in stress scores 19.4 (SD5.7) vs 15.2 (SD6.4), *p* = 0.008).

### Subjective experience

We also surveyed the respondents as to whether they felt more or less time should be dedicated to the longitudinal mindfulness curriculum and whether sessions should be mandatory, elective or a mix. Regarding time spent on the curriculum (currently 10.5 hours, plus drop in opportunities), 6% felt that it should be decreased, 55% that it should stay the same and 38% that it should be increased (1% did not respond). In addition, 29% felt that the curriculum should be elective, 17% mandatory, and 54% a combination elective and mandatory sessions (1% did not respond). Program evaluations were collected throughout the three years of study. While not a primary objective of the current study, they revealed the curriculum to be generally well-received by students, with 87% of feedback being positive to very positive (e.g. “excellent and very important session,” “I always look forward to the mindfulness sessions,” “practical,” “[the teacher] took us through mindfulness exercises that I could see myself incorporating into my life,” “incredibly valuable,” “helped me better understand the application [of mindfulness] throughout my schooling,” “introduced me to something I probably wouldn’t have found [otherwise],” “really a life changer,” “I have already seen improvements in my happiness by living in the present,” “I absolutely love that this content is part of our curriculum,” “am glad to see how much uOttawa cares about student wellness”). Students indicated they enjoyed having the sessions facilitated by clinicians, as they felt it provided role modeling. Thirteen percent of the comments were negative (e.g. “dull,” “I do not find these sessions to be helpful,” “should not be mandatory,” “shallow methods that help decrease stress but in no way address the root causes of higher stress and anxiety in medical schools”). Students also indicated the lunchtime timing of sessions conflicted with student interest group meetings. Teacher evaluation scores averaged 4.6 out of 5.0 (range 4.0-5.0 out of 5.0). There was a larger quantity of feedback from the mandatory sessions than the elective sessions (as expected). Both mandatory and elective sessions were well-received.

## Discussion

To those who are unfamiliar with mindfulness practice, it may be unclear how its regular application, formally and informally, might impact wellbeing. In mindfulness practice, the practitioner attempts to attend to present moment experiences without judgment and with full awareness, curiosity, and compassion.^14^ While the practice does not necessarily change external reality, the practitioner can learn to *relate to* and *experience* reality differently. Mindfulness practice fosters the ability to observe experiences as they are, without the added distortion of thoughts (beliefs, judgement, preconceptions etc.) and emotions which may influence the perception of the experience. This may be a valuable skill when thoughts and emotions are negative or afflictive, and this, in turn, may lead to reduced stress and improved resilience.^14^

Some critics of mindfulness have voiced concerns that this practice implies that the source of unhappiness is ‘all in our heads’ and worry that the practice suggests we accept our circumstances helplessly without attempts to advocate for positive change.^44^ It is imperative to recognize, however, that the ability to observe our present circumstances as they are does not preclude the ability to act in order to change those circumstances moving forward. On the contrary, through the act of observing a situation as it is, without distortion, the practitioner can identify appropriate courses of action that may address the true needs of the situation. Action can be taken in a thoughtfully responsive rather than an emotionally reactive fashion. Furthermore, mindfulness does not displace responsibility for dissatisfying and stressful conditions onto the individual and does not release responsibility from the systems and society in which we work and live. System-level changes to address substandard conditions, inequities, unfairness etc.; and those to promote and support wellbeing will always be needed. While developing curricular interventions to preserve physician wellness, medical schools and healthcare systems are not absolved of their responsibility to address underlying contributors to strain in medical training and practice.

In addition to system-level changes, early exposure to and increased opportunities to participate in wellness-related interventions are warranted, taking into account the high stress and burnout rates compared with the non-medical population.^1,2^ The allure of mindfulness initiatives as part of such a curriculum comes from the essence of this practice nurturing an inherently fundamental human quality - that of compassionate attention. Furthermore, individual mindfulness practice, not solely or necessarily instructor-led sessions, may result in benefits over time.^18^ Once taught, learners can continue to develop their own practice and therefore have a measure of control over their own wellness. Faculties may provide opportunities within the learning and working environment to practice formally, as well to promote the culture change that is necessary to prevent burnout and promote resilience. Our curriculum was developed with these matters in mind; however, it remains modifiable and can evolve over time depending on student and faculty identified successes, weaknesses, opportunities, and barriers.

The analysis of the results from our student population suggests some interesting findings and correlations. First, empathy scores in our students were similar to published scores from other medical student populations, including the slightly higher empathy scores seen in our female respondents. The latter may speak to gender differences in the perception of the emotional experience of others, either biologically programmed or learned.^37,38^ While responses from clerkship were low, and therefore must be interpreted with caution, the empathy scores of our students showed a statistically significant drop between pre-clerkship and clerkship consistent with previous studies on medical student stress and empathy.^8,9,45^ Second, our students’ perceived stress scores were higher than population data (stress scale score of 19.2 overall in our students vs 14.2 for ages 18-29 and 13.0 for ages 20-44 in the general population)^43^, as well as marginally higher than another Canadian medical student population cohort (17 for females and 15 for males).^46^ Furthermore these higher perceived stress scores occurred in the context of relatively low resilience scores compared with population data (resilience scale score of 71.2 in our respondents vs 80 for another first year medical student cohort,^40^ 80.4 for general population, 71.8 for outpatient primary practice setting and 68.0 for outpatient psychiatry setting).^47^ We should hope instead that resilience, defined as the capacity to respond to stress in a healthy way such that goals are achieved at minimal psychological and physical cost,^48^ could be bolstered during times of stress in order to prevent burnout. Burnout has been shown to be related to less altruistic professional values^49^ and poor patient outcomes.^50^ These results require further exploration.

Third, our cross-sectional results show that the degree of mindfulness is positively correlated with empathy and resilience, and negatively correlated with the degree of perceived stress, which is similar to the positive effects associated with mindfulness in the literature.^16,17,23–28^ While correlations cannot be assumed to be causations, it is possible that interventions that increase mindfulness, may have a positive impact on empathy, resilience and stress in medical trainees.

Finally, while the implementation of this curriculum did not affect overall levels of mindfulness, empathy, resilience, or stress scores in our respondents over the three-year study duration, results from students who subjectively reported that they *actively applied* the principles of the curriculum reported statistically higher levels of mindfulness, empathy, and resilience scores and a trend toward lower perceived stress scores compared with students who felt they did not apply the curriculum. Additionally, increased home mindfulness practice was associated with proportionate benefits to mindfulness, empathy, resilience and stress scores that were significant at two hours of practice per week (less than 20 minutes per day). While again these are associations without clear determination of causation, it would be interesting to explore whether or not the beneficial effects of mindfulness practice on empathy, resilience, and stress may be limited to those who practice regularly and may be stronger in those who practice more. These findings make sense if one views mindfulness practice similar to exercise i.e. knowledge of the benefits of the practice will have little impact if there is no regular practice itself. It is only when that knowledge is applied and practiced regularly that results are seen.

In its third year, our core longitudinal mindfulness curriculum was modified to be fully mandatory, as we postulated that expanding the opportunities for students to be exposed to and practice mindfulness regularly as part of their ongoing core curriculum might magnify the benefits seen. While controversial, this was done as a pilot with student support, recognizing that motivation may be required for benefits to be seen^29^ and that imposing wellness initiatives on students raises questions about autonomy. While attendance of the sessions was expected, students were not mandated to participate in the mindfulness practice themselves. Further experience with a mandatory curriculum is needed to fully assess its feasibility, along with any specific successes and challenges. Qualitative research may help clarify the impact of and barriers to such a curriculum and illuminate reasons for low participation in elective curricular components.

## Study limitations

There are several limitations to this study. First, it is possible that the inventory questionnaires may not be ideally suited to accurately quantify the variables under study, or in this particular population. All four scales chosen have, however, been used in other studies involving the medical student population^40,46,52–54^ and when a medical student version of a scale was available (i.e.: Jefferson Scale of Physician Empathy), it was used to target this demographic.

The biggest limitation of this study was the low overall response rate. Response rates were lower from males compared to females and from students in Years 3 and 4 of medical school compared to those in Years 1 and 2. Study results therefore cannot be generalized to the entire student population, and particularly might not be representative of students in the higher years of study or of the male student population. Probable reasons for reduced response rate in clerkship years include reduced time due to the focus on clinical duties, the relative paucity of mindfulness exposure in clerkship (single workshop) compared with the pre-clerkship years (eight sessions) and the lack of interest in the subject matter. When the response rate from pre-clerkship students alone is considered, our data is somewhat more robust. At present, this is the group to whom the bulk of the mindfulness curriculum is delivered.

Two other major study limitations are potential selection bias and lack of paired responses over the three-year study period as students moved through the curriculum. Interestingly, the survey response rate mirrored the student participation rates for the elective sessions. It is possible that the students participating in the study were inherently more interested in the subject matter and more open to the practice than non-responders. It is also unknown whether respondents were more or less stressed or more or less resilient than non-responders. The responses to the study questionnaires at all four time points were not paired and therefore changes in the measured scales within an individual over time cannot be inferred. This is a significant limitation in identifying any potential impact of the curriculum over time. Given the overall low response rate, it is difficult to know if there would have been any interpretable data from paired sets. Instead, group changes over time were analyzed.

The possibility of recall error also exists when estimating application of the concepts of the curriculum and amount of home practice. Accurately quantifying measures such as these is challenging. Furthermore, while mindfulness practice done outside of the curriculum could be perceived as a confounder, the participation in home and other group mindfulness practice outside of the curriculum is encouraged; indeed, in the opinion of the authors, one of the goals of the curriculum is to stimulate student interest to pursue contemplative practice.

Lastly, as previously stated, correlation results need to be interpreted with care, as none of the correlations imply causality.

While these preliminary findings do need to be interpreted with caution, our results have been very useful for ongoing program development. Some of the lessons learned and our curriculum structure may be useful to other faculties of medicine wishing to add contemplative practice education into their core medical school curricula in order to foster wellness-related outcomes. For example, ways to promote home mindfulness practice and personal application of mindfulness principles should be considered. Explicitly linking mindfulness practice to improved clinical skills, such as communication, clinical assessment, and more educated patient referrals for this treatment modality, may be another means of encouraging student engagement with the practice. Further experience and research will continue to inform the best method of curriculum delivery (mandatory versus elective versus combination) in our local setting and whether a more intensive curriculum targeted at specific ‘at-risk’ time points will better address student needs.

### Conclusion

The implementation of a longitudinal mindfulness curriculum in undergraduate medical education was feasible and was well received by these students. Despite this, attendance at elective sessions was low and the reasons for this requires further exploration. Compared to population norms, stress level scores in our respondents were high and resilience scores relatively low throughout the course of medical school training. Mindfulness scores correlated positively with those of empathy and resilience and negatively with perceived stress. Respondents who felt they implemented the principles of the curriculum had higher empathy and resilience scores and a trend toward lower stress scores. The amount of home practice appeared to influence the size of these effects. Further study is warranted to determine if a formal mindfulness curriculum and support of home mindfulness practice in medical students are worthwhile.
